# Evaluation of phenoxybenzamine in the CFA model of pain following gene expression studies and connectivity mapping

**DOI:** 10.1186/1744-8069-6-56

**Published:** 2010-09-16

**Authors:** Meiping Chang, Sarah Smith, Andrew Thorpe, Michael J Barratt, Farzana Karim

**Affiliations:** 1 Indications Discovery Research Unit, Pfizer Global Research and Development, 700 Parkway West, Chesterfield, MO, 63017, USA; 2 Inflammation Research Unit, Pfizer Global Research and Development, 700 Parkway West, Chesterfield, MO, 63017, USA

## Abstract

**Background:**

We have previously used the rat 4 day Complete Freund's Adjuvant (CFA) model to screen compounds with potential to reduce osteoarthritic pain. The aim of this study was to identify genes altered in this model of osteoarthritic pain and use this information to infer analgesic potential of compounds based on their own gene expression profiles using the Connectivity Map approach.

**Results:**

Using microarrays, we identified differentially expressed genes in L4 and L5 dorsal root ganglia (DRG) from rats that had received intraplantar CFA for 4 days compared to matched, untreated control animals. Analysis of these data indicated that the two groups were distinguishable by differences in genes important in immune responses, nerve growth and regeneration. This list of differentially expressed genes defined a "CFA signature". We used the Connectivity Map approach to identify pharmacologic agents in the Broad Institute Build02 database that had gene expression signatures that were inversely related ('negatively connected') with our CFA signature. To test the predictive nature of the Connectivity Map methodology, we tested phenoxybenzamine (an alpha adrenergic receptor antagonist) - one of the most negatively connected compounds identified in this database - for analgesic activity in the CFA model. Our results indicate that at 10 mg/kg, phenoxybenzamine demonstrated analgesia comparable to that of Naproxen in this model.

**Conclusion:**

Evaluation of phenoxybenzamine-induced analgesia in the current study lends support to the utility of the Connectivity Map approach for identifying compounds with analgesic properties in the CFA model.

## Background

Several recent studies have characterized preclinical pain models with the goal of defining gene expression profiles related to different kinds of pain [1-9]. An understanding of gene alterations associated with pain is important as these may open up new pathways for targeting pain. While there are studies that describe gene expression profiles following nerve transection or nerve ligation models in animals [1-5,9], reports defining gene expression in studies that utilize inflammatory models such as the Complete Freund's Adjuvant (CFA) model are scarce. The rat CFA model yields acute inflammation and pain as measured by tactile allodynia [10-12], changes in weight bearing, and paw pressure withdrawal thresholds [13]. We have used this model for screening potential compounds for osteoarthritic pain [11-13]. The non-steroidal anti-inflammatory drug (NSAID), naproxen [14], is active in the CFA model, reducing mechanical allodynia, weight bearing differentials and increasing the paw pressure withdrawal thresholds and is thus a clinically relevant positive control for this model.

The Connectivity Map was recently described by Lamb et al [15], as an approach to identify connections between diseases and drugs, based on their gene expression signatures. This resource consists of a large public database that includes hundreds of compound profiles termed the "reference gene signatures" obtained from cultured human cell lines treated with more than thirteen hundred pharmacologically active agents (Broad Build02 database) [15]. The database is attached to a pattern-matching tool. Gene signatures of interest from expression profiling studies can be compared to the Connectivity Map database, and subjected to a pattern matching algorithm that ranks similarities between the gene signature of interest and the reference signatures [15]. This can lead to identification of 'connections' between an investigator's gene expression signature (e.g. a disease tissue/model) and small molecule profiles in the database. Several recent studies have used this resource to identify novel agents for different indications, including hair growth and leukemia [16,17].

Dorsal Root Ganglia (DRG) are known to be important sites for pain processing. In this study, we first generated gene expression profiles of the ipsilateral L4 and L5 dorsal root ganglia (DRG) extracted from rats injected with CFA in their left paws for 4 days and compared them to profiles of L4 and L5 DRGs from naïve rats. The gene signature of CFA-treated versus naïve rats was created and queried in the Connectivity Map database. Phenoxybenzamine - an alpha adrenergic receptor antagonist and several other agents showed significant inverse connectivity to the CFA signature. We thus reasoned that these agents could potentially reduce pain processing and have antinociceptive properties in the CFA model. Here we describe the data leading to the generation of this hypothesis and our subsequent experimental confirmation of the activity of phenoxybenzamine in this model of inflammatory pain.

## Methods and materials

### Animals

Adult Male Sprague Dawley rats (200-250 g) were used in all experiments. Rats were purchased from Harlan, (Indianapolis, IN) and maintained on a 12/12 hr light/dark cycle with food and water ad libitum. Rats were acclimated for a week before use in experiments. The Pfizer Institutional Animal Care and Use Committee reviewed and approved the animal use in these studies. The animal care and use program is fully accredited by the Association for Assessment and Accreditation of Laboratory Animal Care, International.

### CFA-induced inflammation

Rats were anesthetized briefly with isoflurane (5% induction, then 2% maintenance) and their left foot swabbed with ethanol. 0.15 ml Complete Freund's adjuvant (CFA, SIGMA, St. Louis, MO) was injected subcutaneously into the plantar surface of the left hind paw of the rat [10]. The CFA injection immediately induces local inflammation, paw swelling and pain, which persists for at least 2 weeks post-injection. For behavior studies, rats were placed on the equipment and left to acclimate for 30 minutes. On day 0, baseline measurements were read and rats were injected with CFA thereafter. On day 3, post-CFA reads were taken and only rats that met criteria of hyperalgesia were placed on the study on day 4.

### Mechanical allodynia and thermal hyperalgesia

To assess mechanical allodynia, rats were placed on an elevated wire mesh platform, and to confine their movement, a 15 × 22 × 25 cm plexiglass chamber was placed over each animal. Mechanical paw withdrawal thresholds (PWT) were measured by using a set of Semmes Weinstein monofilaments (Stoelting, Wood Dale, IL) using the Dixon up and down method [18]. Only rats that displayed a PWT of 8 g or less on day 3 (post-CFA) were placed on study. To assess thermal hyperalgesia rats were placed on glass plates with the source of heat applied from the bottom. On day 3 (post-CFA) rats that gave withdrawal latencies of 6 s or less were included in the experiments. Rats were then randomly assigned to either a vehicle group or drug group. On day 4, rats were treated with either the vehicle (saline), or drug and reads were taken 2 hrs after the treatment. All measurements were performed fully randomized and blinded and the reader did not know what treatment each rat received.

### Drug administration

All drugs/vehicle were administered by oral gavage at 5 mg/kg/ml. Naproxen and phenoxybenzamine hydrochloride were purchased from SIGMA (St. Louis MO). Both were dissolved in saline, which was used as vehicle. All measurements were taken 2 hr post dosing. A group of naïve rats was also used in experiments, and these were not injected with CFA, and were not dosed.

### Statistical analysis of behavioral data

This was done on the raw data using a one way ANOVA followed by the Student- Newman-Keul's Post-Hoc test.

### RNA isolation and gene array profiling

A naive (control) group of rats (n = 6) and a group of rats injected with CFA (n = 6) as described above, were used for gene expression profiling experiments. On day 4, rats were euthanized and ipsilateral L4 and L5 dorsal root ganglia (DRG) were extracted, pooled for each rat, and immediately frozen in liquid nitrogen. DRG were stored at -80°C until used. RNA isolation and processing for gene arrays was done according to standard protocols by Genelogic (Boston, MA). Briefly, dorsal root ganglia tissues were homogenized in guanidium isothiocyanate with β-mercaptoethanol (GITC+BME), using the Omni TH-115 homogenizer. Trizol was added and mixed by repeated inversions. RNA aqueous phase was separated using Heavy Phase Lock Gel tubes. Chloroform and sample homogenate were added to the tube and shaken vigorously to remove protein contaminants, incubated on ice for 9-12 min, then centrifuged. The aqueous phase was cleaned using the Qiagen RNEasy Mini Kit (Cat # 74106). A bead cleanup was performed using Agencourt RNAClean magnetic beads (Cat # A29168) to further purify the RNA. RNA quality was evaluated by an Agilent Bioanalyzer. Only the RNA samples that passed the quality control measures proceeded for microarray. 12 high quality RNA samples each from an individual animal were prepared. 1 RNA sample from an animal in the control group had low yield and did not get processed further. The remaining 11 RNA samples were each run on a separate microarray. All 11 microarrays passed the quality control criteria and proceeded to downstream analysis. In total, 5 microarrays each from the 5 animals in the control group, and 6 microarrays each from the 6 animals in the CFA group were analyzed.

### Rat whole genome 4X44K one color microarray processing

Sample preparation, hybridization, wash, scanning and feature extraction was performed according to the standard protocol, 'One-Color Microarray-Based Gene Expression Analysis v.5.7 (P/N G4140-90040)', by Agilent Technologies. One exception was that Triton was excluded from the wash buffer. Only the microarrays that passed the quality control criteria proceeded to downstream analysis.

### Microarray data processing

The Agilent rat whole genome microarray signals were normalized with a program developed internally. Briefly, signal normalization against a common sample was performed using a smoothed piecewise linear curve, trained on the log intensities that differed by < 10% rank order between two samples. Local background subtraction was performed on each array prior to normalization. A computational naïve control pool was created from the averaged normalized signals of the individual naïve control arrays. Within the naïve control arrays, a median array was selected as the one least distant from the other naïve control arrays. All naïve control arrays were normalized against the median array prior to calculating the averaged normalized signals of the control pool. Finally each sample was normalized against the computational naïve control pool. Fold changes between samples were calculated, raising weak signals to a minimum value of 10 prior to comparison. Fold and p-values from student's t-test between groups was calculated.

### Connectivity Map

The Broad's Connectivity Map algorithm was implemented in-house. All compound profiles were downloaded from Broad Build02 database [15]. A nonparametric, rank-based pattern matching strategy based on Kolmogorov-Smirnov statistics was devised and used in the calculation of the Connectivity Score and p-value as described in [15] and [19].

### Gene Expression Pathway Analysis

Oligo probes that met the fold and p-value cutoff (arbitrarily set at 1.5 fold increase or decrease and p-value < = 0.05) were used for Functional Analysis using Ingenuity Pathway Analysis (Ingenuity IPA 8.0 (content version 2602); Ingenuity^® ^Systems, http://www.ingenuity.com). Functional Analysis identified the biological functions and/or diseases that were significant to the dataset. Fischer's exact test was used to calculate a p-value determining the probability that each biological function and/or disease assigned to that dataset is due to chance alone. Canonical Pathway Analysis identified the pathways from the Ingenuity Pathways Analysis library of canonical pathways that were significant to the dataset. The significance of the association between the data set and the canonical pathway was measured in 2 ways: 1) A ratio of the number of genes from the dataset that map to the pathway divided by the total number of genes that map to the canonical pathway is displayed. 2) Fisher's exact test was used to calculate a p-value determining the probability that the association between the genes in the dataset and the canonical pathway is explained by chance alone.

### Hierarchical Clustering

The transcript expression signal intensity was first mean centered and normalized across samples and then a complete linkage clustering method was applied on the correlation similarity measure for hierarchical clustering and heat map in Spotfire^® ^DecisionSite.

## Results

### CFA-behavior studies

**Figure 1:**
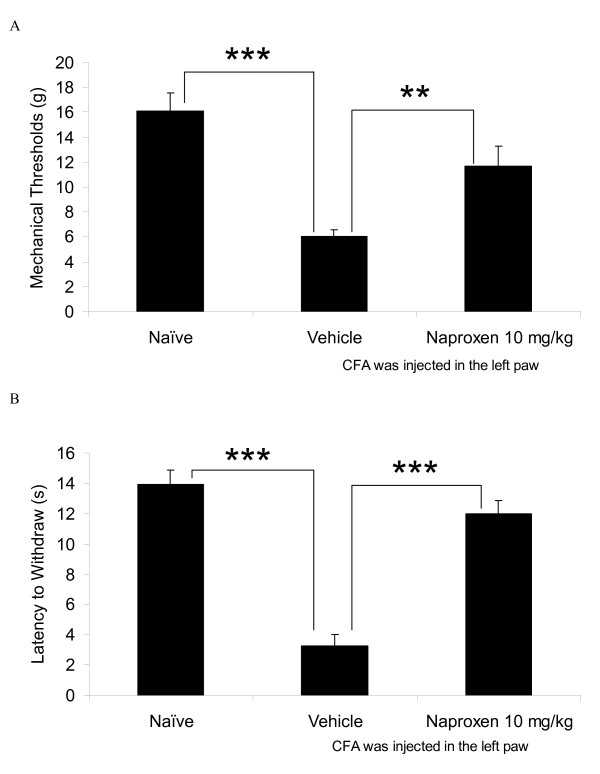
**Oral naproxen is effective in reducing nociceptive behavior in 4 day CFA-injected rats**. Naproxen reduced A) mechanical thresholds and B) paw withdrawal latencies taken 2 hr post dose. n = 6-16 rats per group. For this and other figures, data is presented as mean +/- s.e.m. **p < 0.01 and ***p < 0.001, significant differences from vehicle (saline) - dosed rats.

Injection of CFA significantly reduced mechanical paw withdrawal thresholds (PWT) by 62% when compared to naïve rats (P < 0.001, Figure 1A). Naproxen (10 mg/kg) significantly reversed mechanical allodynia back to about 73% (P < 0.01, Figure 1A) of naïve mechanical thresholds. Injection of CFA significantly induced thermal hyperalgesia as measured by a decrease of latencies to withdraw by about 76% when compared to naïve rats (P < 0.001, Figure 1B). Naproxen significantly reversed the thermal hyperalgesia back to about 86% of naïve controls (P < 0.001, Figure 1B). These effects are in close agreement with previously reported data [11,12].

### Gene expression microarray studies

**Figure 2:**
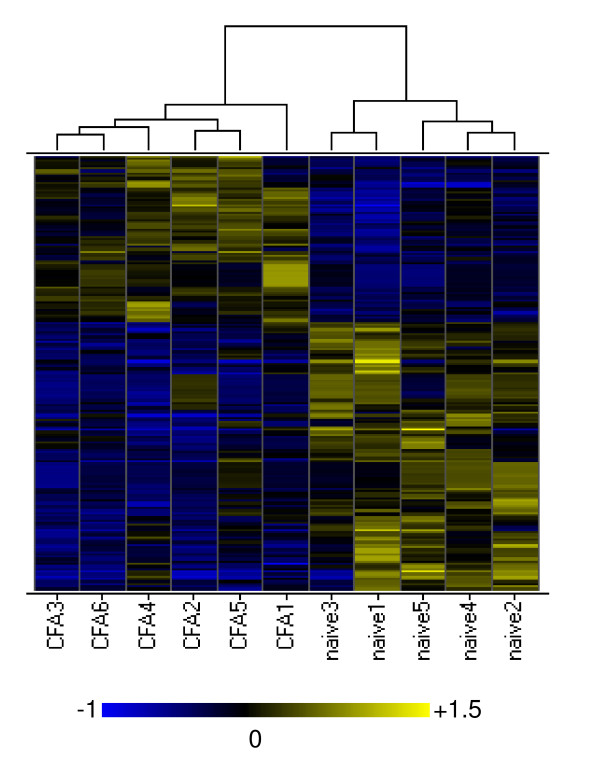
**Heat map of transcripts differentially expressed in the DRG between naïve and CFA injected rats**. The branch length in the dendrogram represents the relation between the samples, the shorter the branch, the higher the similarity between the samples. Up-regulated genes are shown in yellow and down-regulated genes are in blue.

Gene expression in DRG was compared between arrays obtained from 4 day CFA-injected rats and from naïve rats. Genes that were up- or down- regulated by ≥ 1.5 fold (p ≤ 0.05), were used to generate a differential expression list for CFA-treated versus naive rat DRG. 235 oligo probes met these criteria. 178 oligo probes (representing 140 unique genes) have HUGO gene symbols and further analysis was focused on these genes. Hierarchical clustering indicated that these transcripts could separate the CFA samples from naïve samples, as shown in the heat map (Figure 2). We thus consider these transcripts as a molecular signature of the 4 day CFA model in the DRG tissue.

**Figure 3:**
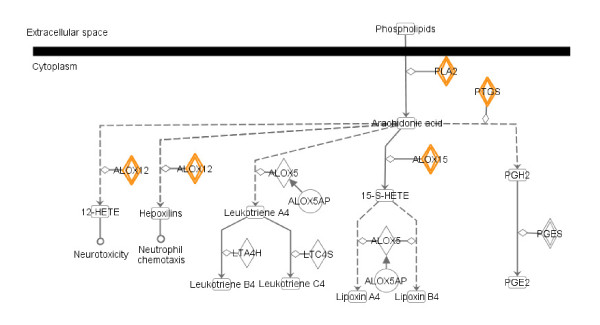
**The eicosanoid signaling pathway from Ingenuity Pathway Analysis software**. Only part of the pathway is shown. The highlighted genes (in yellow) were down-regulated in the 4 day CFA model of inflammatory pain. PLA2: phospholipase A2 group IVA; PTGS: prostaglandin-endoperoxide synthase 1; ALOX12, ALOX15: rat arachidonate 12/15-lipoxygenase.

Overall, it appears that genes that mediate inflammation are suppressed, while genes that mediate neuron growth/survival are enhanced in this 4 day CFA gene signature. Ingenuity^® ^Functional and Pathway Analysis (IPA) was conducted on the genes decreased by CFA treatment in the DRG to help understand the function and canonical pathways perturbed. IPA is a web-based application that enables the visualization, discovery and analysis of molecular functions and pathways within gene expression profiles. One function identified that was significantly affected (p-value < 0.01) is suppression of a group of genes that have a role in immune response or inflammatory disorders (Table 1). Also noted is that leucine rich repeat interacting protein 1 (Lrrfip1), a gene that represses TNF alpha expression, is increased in the DRG from CFA-treated rats compared to that from naïve rats. This data suggests that the immune and inflammatory response may play a role in regulating pain processing in the CFA treated DRG. The eicosanoid signaling pathway was also significantly affected in this model (p-value = 4E-3), with multiple pathway components down-regulated in the DRG tissue of the CFA-treated group 4 days after CFA treatment (Figure 3). These include the phospholipase A2 group IVA (PLA2g4a), prostaglandin-endoperoxide synthase 1 (Ptgs1), and rat arachidonate 12/15-lipoxygenase. In addition, several genes that might modulate the release of eicosanoids, such as BCL6 [20], BTK [21] and CNR2 [22], were also reduced 4 days post-CFA injection.

**Table 1:** Immune and inflammatory response genes down-regulated in the DRG by intraplantar CFA

Gene Symbol	Gene Name	Fold
Adamts12	ADAM metallopeptidase with thrombospondin type 1 motif, 12	-1.6
Alox15	arachidonate 15-lipoxygenase	-2.1
Bcl6	B-cell CLL/lymphoma 6	-1.6
Btk	Bruton agammaglobulinemia tyrosine kinase	-1.5
Ca6	carbonic anhydrase VI	-1.6
Casp8	caspase 8, apoptosis-related cysteine peptidase	-1.5
Cd22	CD22 molecule	-2.2
Cd52	CD52 molecule	-1.9
Cnr2	cannabinoid receptor 2 (macrophage)	-1.7
Colec12	collectin sub-family member 12	-1.9
Cpa3	carboxypeptidase A3 (mast cell)	-2.4
Ctgf	connective tissue growth factor	-1.6
Ctse	cathepsin E	-1.8
Cybb	cytochrome b-245, beta polypeptide	-1.6
Efna4	ephrin-A4	-1.5
F13a1	coagulation factor XIII, A1 polypeptide	-1.8
Fgr	Gardner-Rasheed feline sarcoma viral (v-fgr) oncogene homolog	-1.5
Hcls1	hematopoietic cell-specific Lyn substrate 1	-1.8
Hecw2	HECT, C2 and WW domain containing E3 ubiquitin protein ligase 2	-1.6
Ikzf1	IKAROS family zinc finger 1 (Ikaros)	-1.8
Il1r2	interleukin 1 receptor, type II	-1.6
Il21r	interleukin 21 receptor	-1.5
Il2rb	interleukin 2 receptor, beta	-1.6
Klra7	killer cell lectin-like receptor, subfamily A, member 7	-1.6
Lox	lysyl oxidase	-1.6
Npas2	neuronal PAS domain protein 2	-1.7
Odz4	odz, odd Oz/ten-m homolog 4 (Drosophila)	-1.5
Pdgfrl	platelet-derived growth factor receptor-like	-1.7
Pla2g4a	phospholipase A2, group IVA (cytosolic, calcium-dependent)	-1.9
Ptgs1	prostaglandin-endoperoxide synthase 1	-1.9
Rara	retinoic acid receptor, alpha	-1.6
Si	sucrase-isomaltase (alpha-glucosidase)	-1.6
Sit1	signaling threshold regulating transmembrane adaptor 1	-1.5
Spag16	sperm associated antigen 16	-1.6
Tnfrsf14	tumor necrosis factor receptor superfamily, member 14	-1.7
Tpsb2	tryptase beta 2	-1.5
Xdh	xanthine dehydrogenase	-1.9

Another group of genes suppressed by CFA encode proteins that may contribute to T cell function. Among these genes, IL21R [23], IL2RB [24], RHOH [25], CNR2 [26], IKZF1 [27], BCL6 [28], PTGS1 [29], CASP8 [30], and CD52 [31] were all down-regulated. In addition, several genes related to T-cell function were increased (Table 2) in the CFA treated rat DRGs. Examples include IL22RA2 [32]; CD7 [33]; Lgals2 (also called galectin-2) [34], and IL2RG [35].

**Table 2:** Genes up-regulated in the DRG by intraplantar CFA

Gene Symbol	Gene Description	Fold
Ccl2	chemokine (C-C motif) ligand 2	1.7
Enc1	ectodermal-neural cortex 1	1.8
Hgf	hepatocyte growth factor	1.5
Nmu	neuromedin U	1.5
Per2	period homolog 2	1.6
Psip1	PC4 and SFRS1 interacting protein 1	1.6
Slc30a3	solute carrier family 30	1.7
Srd5a2	steroid-5-alpha-reductase, alpha polypeptide 2	1.7
Zhx2	zinc fingers and homeoboxes 2	1.6
Cacna1d	calcium channel, voltage-dependent, L type, alpha 1D subunit	1.5
Cd7	Cd7 molecule	2.7
Defb50	defensin beta 50	1.6
Dennd4b	similar to brain specific protein 4	1.5
Dnah1	dynein, axonemal, heavy chain 1	1.5
Foxred2	similar to hypothetical protein FLJ23322	1.5
G1p2	interferon, alpha-inducible protein (clone IFI-15K)	1.5
Gmcl1	germ cell-less homolog 1	1.6
Hmgn2	high mobility group nucleosomal binding domain 2	1.6
Hnf4a	hepatocyte nuclear factor 4, alpha	1.8
Hpse2	heparanase 2	1.6
Htr2b	5-hydroxytryptamine (serotonin) receptor 2B	2.1
IgG-2a	gamma-2a immunoglobulin heavy chain	1.5
Il22ra2	interleukin 22 receptor, alpha 2	1.7
Il2rg	interleukin 2 receptor, gamma	1.5
Klhl34	similar to hypothetical protein FLJ34960	1.6
Klk9	kallikrein related-peptidase 9	1.7
Lgals2	lectin, galactoside-binding, soluble 2	1.5
Lrrfip1	leucine rich repeat (in FLII) interacting protein 1	1.6
Muc4	mucin 4, cell surface associated	1.6
Olr1092	olfactory receptor 1092	1.5
Olr1229	olfactory receptor 1229	1.6
Olr1366	olfactory receptor 1366	1.5
Olr233	olfactory receptor 233	1.8
Olr327	olfactory receptor 327	1.8
Olr651	olfactory receptor 651	1.6
Olr859	olfactory receptor 859	1.9
Podxl	podocalyxin-like	2.4
Pole4	polymerase (DNA-directed), epsilon 4 (p12 subunit)	1.6
Pot1b	similar to Protection of telomeres 1	1.9
Ppp1r8	protein phosphatase 1, regulatory (inhibitor) subunit 8	1.5
Rnf43	ring finger protein 43	1.5
Scn7a	sodium channel, voltage-gated, type VII, alpha	1.6
Sele	selectin, endothelial cell	2.1
Spetex-2F	Spetex-2F protein)	1.7
Stac2	SH3 and cysteine rich domain 2	1.6
Tm4sf20	transmembrane 4 L six family member 20	1.7
Tmbim4	transmembrane BAX inhibitor motif containing 4	1.6
Tmprss3	transmembrane protease, serine 3	1.5
Tmprss8	transmembrane protease, serine 8 (intestinal)	1.7
Ttll3	similar to tubulin tyrosine ligase-like family, member 3	1.6
Vom2r40	vomeronasal 2 receptor, 40	1.5

Genes that were up-regulated in the DRG of CFA treated rats, include some that have been previously shown to play a role in pain and neuronal growth, differentiation and prevention of neuronal death. These include neuromedin U, heptocyte growth factor, steroid-5-alpha-reductase (SRD5A2), SLC30A3, PC4, SFRS1 interacting protein 1 (PSIP1), PER2, ZHX2 and ENC1. The functions of these are discussed in the discussion section.

### Connectivity Map Results

**Figure 4:**
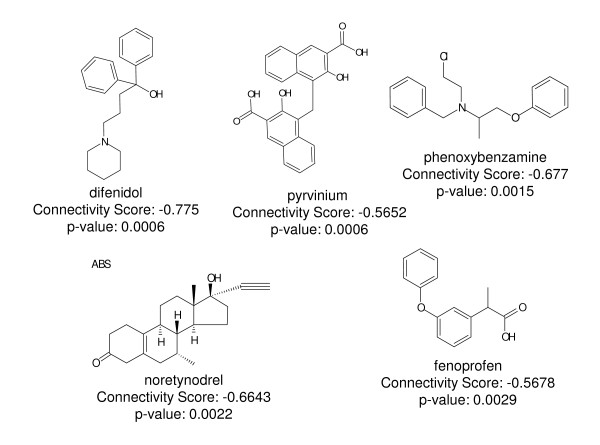
**Compounds identified following connectivity mapping of the CFA signature**.

Using the list of differentially expressed genes between the CFA-treated and naive rats as a DRG CFA signature, we evaluated an internal version of the Broad's Connectivity Map to identify compounds that display inverse connectivity with this CFA signature. This approach yielded five compounds that met the criteria with a p-val < 0.005 and a connectivity score < -0.5 (Figure 4). Based on their negative connectivity with the CFA signature, we hypothesized that these compounds could potentially reduce pain in the CFA model. To test this further, we selected one of the most strongly inferred compounds, phenoxybenzamine, for follow up experiments in the 4 day CFA model to examine its antinociceptive potential.

### Phenoxybenzamine in the 4 day CFA assay

**Figure 5:**
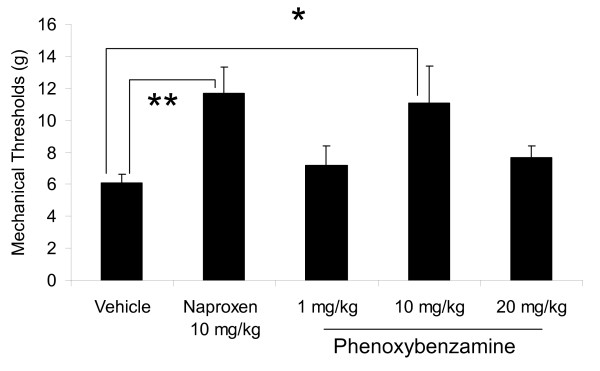
**Effect of oral phenoxybenzamine administered to rats on day 4 of the CFA model of inflammatory pain**. Mechanical thresholds were read 2 hours post-dosing. n = 8-15 per group. *p < 0.05 and **p < 0.01, significant differences from vehicle (saline) - dosed rats.

Phenoxybenzamine was evaluated in the 4 day CFA model using mechanical allodynia as an outcome measurement and with naproxen as a positive control. When compared to the vehicle treated rats, naproxen (10 mg/kg) reversed mechanical allodynia by 93% (P < 0.01, Figure 5). Phenoxybenzamine, at 10 mg/kg was almost as efficacious as naproxen and reversed mechanical allodynia by 82% (P < 0.05, Figure 5).

## Discussion

In this study, we first conducted gene expression microarray experiments to examine transcriptional profiles in the ipsilateral dorsal root ganglia (DRG) of adult rats in a 4 day CFA model of inflammatory pain and generated a rat CFA model gene signature. Our second aim was to identify compounds that were negatively connected to this CFA gene signature using the Connectivity Map approach. Our third objective was to test the hypothesis that compounds identified in this way could reduce CFA-induced pain in this 4 day model. Reports on transcriptional profiling in inflammatory pain models are scarce. Yang et. al. examined gene expression changes in the inflamed tissues and the corresponding DRGs in a carrageenan model using a mini-array containing 100 cytokines, chemokines and related receptors [8]. Parkitna et. al. conducted transcriptional profiling of 4000 genes in the lumbar section of rat spinal cord of a 3-day and a 14-day CFA model [4]. The authors found a dramatic shift in the regulation of secretory vesicle trafficking in the spinal cord in the CFA model. The authors did not profile the L4-L5 DRG. However, they selected 4 transcripts that were changed in the spinal cord and tested them in the L4-5 DRG with qPCR. Consistent with our results, none of the 4 transcripts were significantly changed in the CFA DRG comparing to the naïve rats. To the best of our knowledge, there are no published DRG whole genome microarray studies following 4 days CFA inflammation in rats. We used the CFA model since commonly used NSAIDs such as naproxen that are used to treat OA pain are effective in this model and it is thus frequently used pre-clinically to identify new therapies for OA pain [11,12].

By clustering differentially expressed genes by function or related pathways, we identified a number of genes that are implicated in the modulation of the immune system. Surprisingly, we found that the expression of these genes is decreased in the CFA-treated rats, suggesting suppression of the immune system at day 4 post-CFA treatment in this model system. These include genes in the eicosanoid signaling pathway, including phospholipase A2 group IVA (PLA2g4a), prostaglandin-endoperoxide synthase 1 (Ptgs1), and rat arachidonate 12/15-lipoxygenase. In addition, several genes such as BCL6, BTK and CNR2 that may modulate release of eicosanoids [20-22] are also reduced in the ipsilateral DRGs four days after CFA treatment. We speculate that these genes are early players that initiate inflammation and pain, and their signals are carried forth by other pathways that maintain chronic pain/inflammation with time.

Another group of genes down-regulated by CFA treatment encode proteins that regulate T cell function. These include IL21 receptor subtype II, IL2 receptor beta, RHOH (Ras homolog gene family member H), cannabinoid receptor 2, IKZF1, CASP8, and CD52 [23-31]. It is also noteworthy that IL22RA2, a soluble receptor for IL22 that antagonizes IL22 which is primarily produced by activated T cells [32], CD7 which promotes T cell apoptosis [33], Lgals2 (galectin-2), a proapoptotic effector of activated T-cells [34], and IL2 receptor gamma [35] are all increased in the CFA group compared to the naïve group. This may represent a mechanism of communication by the DRG that modulates T cell activity during inflammation or pain processing. In this regard, T cells have been recently suggested to play a role in neuropathic pain [36,37]. In a model where DRG inflammation was induced by administration of epidural zymosan in incomplete Freund's Adjuvant [7], an increase of CCL2 (MCP-1) among other cytokines was observed, while IL-2 and IL-12 were decreased on day 3 post inflammation. Similarly, in a peripheral inflammation model induced by hind paw injection of carrageenan, both the Scya2 (the CCL2 mRNA) and the gene product MCP-1 (CCL2) were up-regulated following nociceptive stimuli [8]. In that study, Scya2 mRNA was increased about 2-fold in the ipsilateral versus contralateral rat DRG and lasted up to 72 hour post carrageenan. Scya2 mRNA was localized to a subpopulation of vanilloid receptor 1 (TRPV1) containing neurons. Stimulation by a TRPV1 agonist, resiniferatoxin increased expression of Scya2 mRNA. These results are very similar to our findings where we found increased expression of the CCL2 gene in the DRG following CFA injection. CCL2 has also been reported to be up-regulated in the DRG in models of neuropathic pain ([38] and [39] for review). Thus, CCL2 seems to be a common signal elicited in different preclinical pain models.

Other genes that are induced in the DRG of CFA treated rats, include some that encode proteins either known to play a role in pain, promote neuronal growth/differentiation, or prevent neuronal death. One that stands out is neuromedin U (NMU) which has been shown to have an emerging physiological role in nociception upon binding to the NMU receptor 2 [40]. Mice deficient in NMUR2 displayed reduced thermal nociceptive responses in the hot plate test, decreased thermal hyperalgesia following capsaicin injection and reduced the late phase response in the formalin test [41]. In other studies, NMU inhibited inflammation-mediated memory impairment and neuronal cell-death in rodents [42]. Ketterer et. al. (2009) have shown recently that NMU may signal via the hepatocyte growth factor (HGF) c-Met pathway. In our study, HGF is also increased in the DRG following CFA treatment. HGF is a pleiotropic cytokine which partly functions to promote neuronal survival and growth [43]. HGF cooperates with nerve growth factor (NGF) to enhance axonal outgrowth from cultured dorsal root ganglion (DRG) neurons. HGF also enhances the neurotrophic activities of NGF in vivo where Met receptor signaling is required for the survival of a proportion of DRG neurons [44].

Another gene which is up-regulated in the DRG is the steroid-5-alpha-reductase (SRD5A2) which is a key enzyme in the conversion of several Δ4-3keto steroids, such as testosterone, progesterone, aldosterone and corticosterone, into their respective reductase derivatives. Morphine has been shown to increase SRD5A2 level and SRD5A2 inhibitor finasteride potentiates the antinociceptive effect of morphine, prevents the development of morphine tolerance in rats, suggesting that SRD5A2 plays a role in pain perception [45]. SLC30A3 which is also increased in the current study is responsible for transport of zinc into synaptic vesicles. It may have a role in neuropathic pain [46].

We also observed a number of genes whose expression in DRG is modulated by CFA, though their roles in nociception have not been established, they may have indirect linkages to pain plasticity. Two genes involved in regulating circadian rhythm are modulated in the DRG of CFA treated rats versus the naïve rats. Pain perception is influenced by the circadian rhythm in humans and in animals [47]. Expression of PER2 is increased, whereas expression of NPAS2 is decreased. Per/Cry form a heterodimer that interacts with the NPAS2/BMAL1 heterodimer to inhibit the transcription of Per and Cry [48]. SFRS1 interacting protein 1 (PSIP1, or Lens epithelium-derived growth factor (LEDGF)) is up-regulated in response to stress and enhances the survival of neurons in the retina and optic nerve [49]. We speculate that the increased expression of PSIP1 we observed may support neuronal growth in the DRG. ZHX2, a transcriptional regulator of neural progenitor cells, is also up-regulated in the DRG of CFA treated rats. Blocking ZHX2 function causes neuronal differentiation, whereas overexpression of ZHX2 or its ephrin-B1 intracellular domain disrupts the normal differentiation of cortical neural progenitor cells [50]. ENC1 (or NRP/B), also a regulator of neuronal differentiation is increased in the DRG of CFA treated rats. Overexpression of NRP/B significantly induced neurite outgrowth in PC12 cells, whereas inhibition of NRP/B by antibodies or siRNA inhibited neurite outgrowth and suppressed the NGF-induced outgrowth of neurites [50,51]. In summary, we have found a number of genes which regulate neuronal growth in the CFA model, suggesting a neuronal growth component to this peripheral inflammation model.

Using the Connectivity Map approach, we identified 5 compounds in the Broad database that were negatively connected with the CFA signature. These compounds belong to different chemical classes and their structural properties are different. They also bind to different targets. Fenoprofen, an NSAID, was among them. Another compound we identified with this approach was pyrvinium, a non-competitive androgen receptor inhibitor [52]. Intrathecal administration of testosterone, an androgen derived from DHEA, has been shown to cause analgesia in neuropathic rats [53]. Difenidol, also identified by Connectivity Mapping is used for treatment of vertigo. The precise molecular target of this drug is not known, although recently it was found to be a ligand for muscarinic receptors: M1, M3 and M4 [54], and may thus play a role in processing of pain stimuli [55].

Phenoxybenzamine, a non-selective *α*-blocker [56] was also identified as having negative connectivity with the CFA pain signature. Previous studies have reported efficacy of phenoxybenzamine in patients with various pain conditions. In one study, 40 patients with causalgia were treated successfully for their pain with phenoxybenzamine [57]. In a case report, 3 out of 4 patients got relief from complex regional pain syndrome type I with phenoxybenzamine [58]. In the current study, we examined effects of phenoxybenzamine in the 4 Day CFA model to determine whether the Connectivity Map approach could identify compounds that had analgesic potential in this model. Our data confirm the antinociceptive properties of phenoxybenzamine; this effect may be due to a direct blockade of adrenergic receptors that may have become supersensitive to catecholamines or perhaps due to possible sympathetic sprouting, as a result of increased expression of nerve growth regulating genes. Sympathetic sprouting in the DRG has been shown to occur in a study that examined gene expression profiles at 3 days post zymosan in incomplete Freund's adjuvant [7]. Furthermore, antinociception has been reported for phenoxybenzamine in the rat hot plate test [59], the mouse tail flick test [60] and mouse writhing tests [61]. Although we do not yet understand the basis for the diminished efficacy of phenoxybenzamine at the higher dose tested here (20 mg/kg), it is possibly due to excessive antagonism of the adrenergic receptors in addition to other mechanisms, such as its agonist-antagonistic properties [60]. Regardless, our data suggest that the CFA pain model may have broader utility for testing compounds that modulate pain involving the sympathetic nervous system.

## Conclusion

We have established a 4 day CFA signature in the DRG that we subjected to a Connectivity Map approach and identified a compound already known to have a role in the treatment of pain, such as fenoprofen. Another of these compounds, phenoxybenzamine, has been reported in the literature to treat complex regional pain syndrome, and our studies confirm the analgesic properties of this compound in the 4 day CFA model. We conclude that use of the Connectivity Map approach to accompany gene expression microarray and behavior studies, represents a potentially fruitful way to identify novel pain therapies.

## Competing interests

The authors declare that they have no competing interests.

## Authors' contributions

MC conceived of the study, analyzed the gene expression microarray data, generated data from connectivity mapping and drafted the manuscript. SS performed part of the behavior studies. AT participated in design of the study. MB participated in design of the study and editing the manuscript. FK conceived of the study, designed and coordinated the study, performed part of the behavior studies, and drafted the manuscript. All authors read and approved the final manuscript.

## References

[B1] Vega-Avelaira D Geranton S Fitzgerald M Differential regulation of immune responses and macrophage/neuron interactions in the dorsal root ganglion in young and adult rats following nerve injury Molecular Pain 2009 5 70 10.1186/1744-8069-5-70 20003309 PMC2799401

[B2] Takeuchi H Kawaguchi S Mizuno S Kirita T Takebayashi T Shimozawa K Torigoe T Sato N Yamashita T Gene Expression Profile of Dorsal Root Ganglion in a Lumbar Radiculopathy Model Spine 2008 33 2483 2488 10.1097/BRS.0b013e318184acc3 18978588

[B3] Costigan M Befort K Karchewski L Griffin R D'Urso D Allchorne A Sitarski J Mannion J Pratt R Woolf C Replicate high-density rat genome oligonucleotide microarrays reveal hundreds of regulated genes in the dorsal root ganglion after peripheral nerve injury BMC Neuroscience 2002 3 16 10.1186/1471-2202-3-16 12401135 PMC139981

[B4] Rodriguez Parkitna JKM Kaminska-Chowaniec D Obara I Mika J Przewlocka B Przewlocki R Comparison of gene expression profiles in neuropathic and inflammatory pain J Physiol Pharmacol 2006 57 14 17033093

[B5] Rabert D Xiao Y Yiangou Y Kreder D Sangameswaran L Segal MR Hunt CA Birch R Anand P Plasticity of gene expression in injured human dorsal root ganglia revealed by GeneChip oligonucleotide microarrays Journal of Clinical Neuroscience 2004 11 289 299 10.1016/j.jocn.2003.05.008 14975420

[B6] Li X Sahbaie P Zheng M Ritchie J Peltz G Mogil J Clark JD Expression genetics identifies spinal mechanisms supporting formalin late phase behaviors Molecular Pain 2010 6 11 10.1186/1744-8069-6-11 20149257 PMC2831877

[B7] Xie WR Deng H Li H Bowen TL Strong JA Zhang JM Robust increase of cutaneous sensitivity, cytokine production and sympathetic sprouting in rats with localized inflammatory irritation of the spinal ganglia Neuroscience 2006 142 809 822 10.1016/j.neuroscience.2006.06.045 16887276 PMC1661830

[B8] Yang HY Mitchell K Keller JM Iadarola MJ Peripheral inflammation increases Scya2 expression in sensory ganglia and cytokine and endothelial related gene expression in inflamed tissue J Neurochem 2007 103 1628 1643 10.1111/j.1471-4159.2007.04874.x 17883394

[B9] Hammer P Banck MS Amberg R Wang C Petznick G Luo S Khrebtukova I Schroth GP Beyerlein P Beutler AS mRNA-seq with agnostic splice site discovery for nervous system transcriptomics tested in chronic pain Genome Res 2010 20 847 860 10.1101/gr.101204.109 20452967 PMC2877581

[B10] Stein CMM Herz A Unilateral inflammation of the hindpaw in rats as a model of prolonged noxious stimulation: alterations in behavior and nociceptive thresholds Pharmacol Biochem Behav 1988 31 155 151 10.1016/0091-3057(88)90372-3 3244721

[B11] Ahn K Johnson DS Mileni M Beidler D Long JZ McKinney MK Weerapana E Sadagopan N Liimatta M Smith SE Lazerwith S Stiff C Kamtekar S Bhattacharya K Zhang Y Swaney S Van Becelaere K Stevens RC Cravatt BF Discovery and characterization of a highly selective FAAH inhibitor that reduces inflammatory pain Chemistry & Biology 2009 16 411 420 10.1016/j.chembiol.2009.02.013 PMC2692831 19389627

[B12] Johnson DS Ahn K Kesten S Lazerwith SE Song Y Morris M Fay L Gregory T Stiff C Dunbar JB Jr Liimatta M Beidler D Smith S Nomanbhoy TK Cravatt BF Benzothiophene piperazine and piperidine urea inhibitors of fatty acid amide hydrolase (FAAH) Bioorganic & Medicinal Chemistry Letters 2009 19 2865 2869 10.1016/j.bmcl.2009.03.080 PMC3150822 19386497

[B13] Cortes-Burgos LA Zweifel BS Settle SL Pufahl RA Anderson GD Hardy MM Weir DE Hu G Happa FA Stewart Z Muthian S Graneto MJ Masferrer JL CJ-13610, an orally active inhibitor of 5-lipoxygenase is efficacious in preclinical models of pain European Journal of Pharmacology 2009 617 59 67 10.1016/j.ejphar.2009.06.058 19580807

[B14] Fendrick AM Greenberg B A review of the benefits and risks of nonsteroidal anti-inflammatory drugs in the management of mild-to-moderate osteoarthritis Osteopathic Medicine and Primary Care 2009 3 1 10.1186/1750-4732-3-1 19126235 PMC2646740

[B15] Lamb J Crawford ED Peck D Modell JW Blat IC Wrobel MJ Lerner J Brunet J-P Subramanian A Ross KN Reich M Hieronymus H Wei G Armstrong SA Haggarty SJ Clemons PA Wei R Carr SA Lander ES Golub TR The Connectivity Map: Using gene-expression signatures to connect small molecules, genes, and disease Science 2006 313 1929 1935 10.1126/science.1132939 17008526

[B16] Ishimatsu-Tsuji Y Soma T Kishimoto J Identification of novel hair-growth inducers by means of connectivity mapping FASEB J 2009 fj.09-145292 20026683 10.1096/fj.09-145292

[B17] Hassane DC Guzman ML Corbett C Li X Abboud R Young F Liesveld JL Carroll M Jordan CT Discovery of agents that eradicate leukemia stem cells using an in silico screen of public gene expression data Blood 2008 111 5654 5662 10.1182/blood-2007-11-126003 18305216 PMC2424160

[B18] Dixon WJ Efficient analysis of experimental observations Annual Review of Pharmacology and Toxicology 1980 20 441 462 10.1146/annurev.pa.20.040180.002301 7387124

[B19] Lamb J Ramaswamy S Ford HL Contreras B Martinez RV Kittrell FS Zahnow CA Patterson N Golub TR Ewen ME A mechanism of cyclin D1 action encoded in the patterns of gene expression in human cancer Cell 2003 114 323 334 10.1016/S0092-8674(03)00570-1 12914697

[B20] Ravaux L Denoyelle C Monne C Limon I Raymondjean M El Hadri K Inhibition of interleukin-1{beta}-induced group IIA secretory phospholipase A2 expression by peroxisome proliferator-activated receptors (PPARs) in rat vascular smooth muscle cells: cooperation between PPAR{beta} and the proto-oncogene BCL-6 Mol Cell Biol 2007 27 8374 8387 10.1128/MCB.00623-07 17908795 PMC2169168

[B21] Kawakami Y Kitaura J Satterthwaite AB Kato RM Asai K Hartman SE Maeda-Yamamoto M Lowell CA Rawlings DJ Witte ON Kawakami T Redundant and opposing functions of two tyrosine kinases, Btk and Lyn, in mast cell activation J Immunol 2000 165 1210 1219 10903718 10.4049/jimmunol.165.3.1210

[B22] Maccarrone M Battista N Centonze D The endocannabinoid pathway in Huntington's disease: A comparison with other neurodegenerative diseases Progress in Neurobiology 2007 81 349 379 10.1016/j.pneurobio.2006.11.006 17276576

[B23] Monteleone G Pallone F Macdonald TT Interleukin-21 (IL-21)-mediated pathways in T cell-mediated disease Cytokine & Growth Factor Reviews 2009 20 185 191 10.1016/j.cytogfr.2009.02.002 19261537

[B24] Liu DV Maier LM Hafler DA Wittrup KD Engineered interleukin-2 antagonists for the inhibition of regulatory T cells Journal of Immunotherapy 2009 32 887 894 10.1097/CJI.0b013e3181b528da 19816193 PMC4078882

[B25] Suzuki HOH The atypical small GTPase RhoH: a novel role in T cell development Nihon Rinsho Meneki Gakkai Kaishi 2008 31 37 46 18311041 10.2177/jsci.31.37

[B26] Maresz K Pryce G Ponomarev ED Marsicano G Croxford JL Shriver LP Ledent C Cheng X Carrier EJ Mann MK Giovannoni G Pertwee RG Yamamura T Buckley NE Hillard CJ Lutz B Baker D Dittel BN Direct suppression of CNS autoimmune inflammation via the cannabinoid receptor CB1 on neurons and CB2 on autoreactive T cells Nat Med 2007 13 492 497 10.1038/nm1561 17401376

[B27] Ronni T Payne KJ Ho S Bradley MN Dorsam G Dovat S Human ikaros function in activated T cells is regulated by coordinated expression of its largest isoforms Journal of Biological Chemistry 2007 282 2538 2547 10.1074/jbc.M605627200 17135265

[B28] Crotty S Johnston RJ Schoenberger SP Effectors and memories: Bcl-6 and Blimp-1 in T and B lymphocyte differentiation Nat Immunol 11 114 120 10.1038/ni.1837 20084069 PMC2864556

[B29] Rocca B Spain LM Puré E Langenbach R Patrono C FitzGerald GA Distinct roles of prostaglandin H synthases 1 and 2 in T-cell development The Journal of Clinical Investigation 1999 103 1469 1477 10.1172/JCI6400 10330429 PMC408457

[B30] Algeciras-Schimnich A Vlahakis SR Villasis-Keever A Gomez T Heppelmann CJ Bou G Paya CV CCR5 mediates Fas- and caspase-8 dependent apoptosis of both uninfected and HIV infected primary human CD4 T cells AIDS 2002 16 1467 1478 10.1097/00002030-200207260-00003 12131184

[B31] Watanabe T Masuyama J-i Sohma Y Inazawa H Horie K Kojima K Uemura Y Aoki Y Kaga S Minota S Tanaka T Yamaguchi Y Kobayashi T Serizawa I CD52 is a novel costimulatory molecule for induction of CD4+ regulatory T cells Clinical Immunology 2006 120 247 259 10.1016/j.clim.2006.05.006 16797237

[B32] Wolk KWE Witte K Warszawska K Sabat R Biology of interleukin-22 Semin Immunopathol 2010 32 17 31 10.1007/s00281-009-0188-x 20127093

[B33] Fukumori T Takenaka Y Yoshii T Kim H-RC Hogan V Inohara H Kagawa S Raz A CD29 and CD7 mediate galectin-3-induced type II T-cell apoptosis Cancer Res 2003 63 8302 8311 14678989

[B34] Sturm A Lensch M Andre S Kaltner H Wiedenmann B Rosewicz S Dignass AU Gabius H-J Human galectin-2: novel inducer of T cell apoptosis with distinct profile of caspase activation J Immunol 2004 173 3825 3837 15356130 10.4049/jimmunol.173.6.3825

[B35] Tsujino S Di Santo JP Takaoka A McKernan TL Noguchi S Taya C Yonekawa H Saito T Taniguchi T Fujii H Differential requirement of the cytoplasmic subregions of γc chain in T cell development and function Proceedings of the National Academy of Sciences of the United States of America 2000 97 10514 10519 10.1073/pnas.180063297 10962026 PMC27056

[B36] Thacker MA Clark AK Marchand F McMahon SB Pathophysiology of peripheral neuropathic pain: immune cells and molecules Anesth Analg 2007 105 838 847 10.1213/01.ane.0000275190.42912.37 17717248

[B37] Costigan M Moss A Latremoliere A Johnston C Verma-Gandhu M Herbert TA Barrett L Brenner GJ Vardeh D Woolf CJ Fitzgerald M T-Cell infiltration and signaling in the adult dorsal spinal cord is a major contributor to neuropathic pain-like hypersensitivity J Neurosci 2009 29 14415 14422 10.1523/JNEUROSCI.4569-09.2009 19923276 PMC2813708

[B38] Gao Y-J Ji R-R Chemokines, neuronal-glial interactions, and central processing of neuropathic pain Pharmacology & Therapeutics 2010 126 56 68 10.1016/j.pharmthera.2010.01.002 PMC2839017 20117131

[B39] White FA Feldman P Miller RJ Chemokine signaling and the management of neuropathic pain Molecular Interventions 2009 9 188 195 10.1124/mi.9.4.7 19720751 PMC2861804

[B40] Mitchell JD Maguire JJ Davenport AP Emerging pharmacology and physiology of neuromedin U and the structurally related peptide neuromedin S British Journal of Pharmacology 2009 158 87 103 10.1111/j.1476-5381.2009.00252.x 19519756 PMC2795236

[B41] Torresa Richard Crolla Susan D Vercollonea Jeffrey Reinhardta Joel Griffithsa Jennifer Zabskia Stephanie Andersona Keith D Adamsa Niels C Gowena Lori Sleemana Mark W Valenzuelaa David M Wieganda Stanley J Yancopoulosa George D Murphya Andrew J Mice genetically deficient in neuromedin U receptor 2, but not neuromedin U receptor 1, have impaired nociceptive responses Pain 2007 130 267 278 10.1016/j.pain.2007.01.036 17379411

[B42] Takashi Iwaia YI Kodania Reiye Oka Jun-Ichiro Neuromedin U inhibits inflammation-mediated memory impairment and neuronal cell-death in rodents Neuroscience Research 2008 61 113 119 10.1016/j.neures.2008.01.018 18336945

[B43] Maina Flavio Hilton Mark C Andres Rosa Wyatt Sean Klein Rüdiger Davies Alun M Multiple roles for hepatocyte growth factor in sympathetic neuron development Neuron 1998 20 835 846 10.1016/S0896-6273(00)80466-3 9620689

[B44] Maina FHM Ponzetto C Davies AM Klein R Met receptor signaling is required for sensory nerve development and HGF promotes axonal growth and survival of sensory neurons Genes Dev 1997 11 3341 3350 10.1101/gad.11.24.3341 9407027 PMC316818

[B45] Verdi J Ahmadiani A Finasteride, a 5[alpha]-reductase inhibitor, potentiates antinociceptive effects of morphine, prevents the development of morphine tolerance and attenuates abstinence behavior in the rat Hormones and Behavior 2007 51 605 610 10.1016/j.yhbeh.2007.02.008 17428486

[B46] Jo SM DG Schrøder HD Suh SW Depletion of vesicular zinc in dorsal horn of spinal cord causes increased neuropathic pain in mice Biometals 2008 151 158 10.1007/s10534-007-9103-x 17570038

[B47] Bruguerolle B Labrecque G Rhythmic pattern in pain and their chronotherapy Advanced Drug Delivery Reviews 2007 59 883 895 10.1016/j.addr.2006.06.001 17716777

[B48] Kitanishi K Igarashi J Hayasaka K Hikage N Saiful I Yamauchi S Uchida T Ishimori K Shimizu T Heme-binding characteristics of the isolated PAS-A domain of mouse Per2, a transcriptional regulatory factor associated with circadian rhythms Biochemistry 2008 47 6157 6168 10.1021/bi7023892 18479150

[B49] Raz-Prag D Zeng Y Sieving PA Bush RA Photoreceptor protection by adeno-associated virus-mediated LEDGF expression in the RCS rat model of retinal degeneration: probing the mechanism Invest Ophthalmol Vis Sci 2009 50 3897 3906 10.1167/iovs.08-3153 19324854 PMC2744960

[B50] Wu C Qiu R Wang J Zhang H Murai K Lu Q ZHX2 interacts with ephrin-B and regulates neural progenitor maintenance in the developing cerebral cortex J Neurosci 2009 29 7404 7412 10.1523/JNEUROSCI.5841-08.2009 19515908 PMC2759685

[B51] Kim T-A Jiang S Seng S Cha K Avraham HK Avraham S The BTB domain of the nuclear matrix protein NRP/B is required for neurite outgrowth J Cell Sci 2005 118 5537 5548 10.1242/jcs.02643 16306221

[B52] Jones Jeremy O B EC Huang Yong Feau Clementine Kiplin Guy R Yamamoto Keith R Byron Hann Marc Diamondabd I Non-competitive androgen receptor inhibition in vitro and in vivo Proc Natl Acad Sci USA 2009 106 7233 7238 10.1073/pnas.0807282106 19363158 PMC2678452

[B53] Kibaly C Meyer L Patte-Mensah C Mensah-Nyagan AG Biochemical and functional evidence for the control of pain mechanisms by dehydroepiandrosterone endogenously synthesized in the spinal cord FASEB J 2008 22 93 104 10.1096/fj.07-8930com 17720801

[B54] Varoli L Andreani A Burnelli S Granaiola M Leoni A Locatelli A Morigi R Rambaldi M Bedini A Fazio N Spampinato S Diphenidol-related diamines as novel muscarinic M4 receptor antagonists Bioorganic & Medicinal Chemistry Letters 2008 18 2972 2976 10.1016/j.bmcl.2008.03.061 18395442

[B55] AM T Muscarinic acetylcholine receptors: new potential therapeutic targets in antinociception and in cancer therapy Recent Pat CNS Drug Discov 2008 3 94 103 10.2174/157488908784534621 18537768

[B56] Frang H Cockcroft V Karskela T Scheinin M Marjamäki A Phenoxybenzamine binding reveals the helical orientation of the third transmembrane domain of adrenergic receptors Journal of Biological Chemistry 2001 276 31279 31284 10.1074/jbc.M104167200 11395517

[B57] Ghostine SY Comair YG Turner DM Kassell NF Azar CG Phenoxybenzamine in the treatment of causalgia Journal of Neurosurgery 1984 60 1263 1268 10.3171/jns.1984.60.6.1263 6726371

[B58] Mario AI Jr Grigory K Treatment of Complex Regional Pain Syndrome Type I with oral phenoxybenzamine: rationale and case reports Pain Practice 2008 8 125 132 10.1111/j.1533-2500.2007.00170.x 18194348

[B59] Cicero TJ Meyer ER Smithloff BR Alpha adrenergic blocking agents: antinociceptive activity and enhancement of morphine-induced analgesia Journal of Pharmacology and Experimental Therapeutics 1974 189 72 82 4150877

[B60] Spiehler VRRL Agonist--antagonist properties of phenoxybenzamine in antinociception and opiate dependence tests Eur J Pharmacol 1979 55 389 395 10.1016/0014-2999(79)90113-4 572775

[B61] Hendershot LC Forsaith J Antagonism of the frequency of phenylquinone-induced writhing in the mouse by weak analgesic and nonalgesics Journal of Pharmacology and Experimental Therapeutics 1959 125 237 240 13642264

